# Microenvironment alters epigenetic and gene expression profiles in Swarm rat chondrosarcoma tumors

**DOI:** 10.1186/1471-2407-10-471

**Published:** 2010-09-01

**Authors:** Christopher A Hamm, Jeff W Stevens, Hehuang Xie, Elio F Vanin, Jose A Morcuende, Hakeem Abdulkawy, Elisabeth A Seftor, Simone T Sredni, Jared M Bischof, Deli Wang, Sergey Malchenko, Maria de Fatima Bonaldo, Thomas L Casavant, Mary JC Hendrix, Marcelo B Soares

**Affiliations:** 1 Interdisciplinary Graduate Program in Genetics, The University of Iowa, Iowa City, IA 52242, USA; 2 Cancer Biology and Epigenomics Program, Children's Memorial Research Center; 3 Department of Orthopaedic Surgery The University of Iowa, Iowa City, Iowa 52242, USA; 4 Robert H. Lurie Comprehensive Cancer Center, Feinberg School of Medicine, Northwestern University, Chicago, Illinois 60614, USA; 5 Department of Pediatrics, Feinberg School of Medicine, Northwestern University, Chicago, Illinois, USA; 6 Department of Electrical and Computer Engineering, The University of Iowa, Iowa City, Iowa, USA; 7 Biostatistics Research Core, Children's Memorial Research Center, Northwestern University's Feinberg School of Medicine, Chicago, Illinois 60614, USA

## Abstract

**Background:**

Chondrosarcomas are malignant cartilage tumors that do not respond to traditional chemotherapy or radiation. The 5-year survival rate of histologic grade III chondrosarcoma is less than 30%. An animal model of chondrosarcoma has been established - namely, the Swarm Rat Chondrosarcoma (SRC) - and shown to resemble the human disease. Previous studies with this model revealed that tumor microenvironment could significantly influence chondrosarcoma malignancy.

**Methods:**

To examine the effect of the microenvironment, SRC tumors were initiated at different transplantation sites. Pyrosequencing assays were utilized to assess the DNA methylation of the tumors, and SAGE libraries were constructed and sequenced to determine the gene expression profiles of the tumors. Based on the gene expression analysis, subsequent functional assays were designed to determine the relevancy of the specific genes in the development and progression of the SRC.

**Results:**

The site of transplantation had a significant impact on the epigenetic and gene expression profiles of SRC tumors. Our analyses revealed that SRC tumors were hypomethylated compared to control tissue, and that tumors at each transplantation site had a unique expression profile. Subsequent functional analysis of differentially expressed genes, albeit preliminary, provided some insight into the role that thymosin-β4, c-fos, and CTGF may play in chondrosarcoma development and progression.

**Conclusion:**

This report describes the first global molecular characterization of the SRC model, and it demonstrates that the tumor microenvironment can induce epigenetic alterations and changes in gene expression in the SRC tumors. We documented changes in gene expression that accompany changes in tumor phenotype, and these gene expression changes provide insight into the pathways that may play a role in the development and progression of chondrosarcoma. Furthermore, specific functional analysis indicates that thymosin-β4 may have a role in chondrosarcoma metastasis.

## Background

Chondrosarcoma is the second most common primary bone malignancy [1] accounting for 25% of primary bone sarcomas [2]. High grade lesions may be treated with chemotherapy/radiation but chondrosarcomas are usually not responsive to treatment [3,4] and, as a result, the 5-year survival rate of histologic grade III chondrosarcoma is only 29% [5].

To attain a greater understanding of chondrosarcoma tumorigenesis, a rat model of human chondrosarcoma has been developed [6-8]. The model, known as the Swarm rat chondrosarcoma (SRC), histologically resembles the human tumor, indicating that the SRC is a suitable model to study chondrosarcoma [7,8]. Experiments with the SRC tumors have demonstrated that transplantation site can affect the malignancy of the tumor, and more specifically, transplantation of the SRC tumor into the tibia results in the formation of a higher grade tumor compared to those derived from extraosseous transplantation [8]. Since tumors grown at different transplantation sites were initiated from the same primary tumor, the increase in malignancy observed with the SRC tibia tumor is likely to result from the interaction between the tumor and its microenvironment.

Although the SRC tumors have undergone extensive histological characterization, no studies have examined the effect that the transplantation site has on epigenetic and gene expression profiles of the SRC tumors.

In this study, tumors were transplanted subcutaneously, or into the tibia of Sprague-Dawley rats. Subcutaneous tumor transplantation led to the formation of significantly larger tumors than those tumors transplanted into the tibia. However, similar to previous SRC experiments [8], transplantation of the SRC tumor into the tibia resulted in the formation of more aggressive tumors that were capable of invading the surrounding bone tissue. SRC tumors were also detected in the lungs of rats that had SRC tumor transplanted into the tibia, but no SRC tumors were detected in the lungs of rats in which tumor cells were injected subcutaneously.

Since changes in DNA methylation can significantly impact SRC tumorigenesis [9], we performed epigenetic analyses to determine the influence that the transplantation environment had on tumor DNA methylation. The analysis revealed that the tumor transplantation site could significantly alter DNA methylation levels in the SRC tumors.

To complement the epigenetic analysis, global gene expression profiles were generated for the SRC tumors using SAGE (Serial Analysis of Gene Expression) [10]. This global gene expression analysis revealed that the SRC tumors have gene expression profiles that are unique to each transplantation site.

Analysis of the differentially expressed genes revealed the pathways that are altered in the SRC tumors, and subsequent functional analyses provided insight into the role that specific genes, namely thymosin-β4, c-fos and CTGF, may play in chondrosarcoma tumorigenesis. Overall, our study highlights the influence of the microenvironment on epigenetic and gene expression profiles of SRC tumors. Such profiles provide an insight into the biological pathways that may be affected by the microenvironment, while underscoring the complex nature of SRC tumorigenesis.

## Methods

### Ethics Statement

Animals were handled in strict accordance with good animal practice as defined by the relevant national and/or local animal welfare bodies, and all animal work was approved by the Institutional Animal Care and Use Committee (Children's Memorial Research Center; protocol IACUC #2006-30).

### Tumor induction and tissue harvesting

The SRC-JWS tumor line (Jeff Stevens, The University of Iowa) was used for all transplantation studies. We demonstrated by microscopy and immunohistochemistry that tumors derived from transplantation of the SRC-JWS tumor line are similar to conventional human chondrosarcoma. Subcutaneous tumors were induced as previously described [11,12]. Briefly, SRC-JWS tumor cells were isolated from a subcutaneous SRC tumor, and 5 × 10^6 ^tumor cells were injected subcutaneously into the lower lumbar region of 4-week-old male Sprague-Dawley rats. In the tibia transplantations, 5 × 10^6 ^SRC-JWS tumor cells were injected into the proximal tibia as previously described [7]. Injection of SRC-JWS tumor cells into the tibia also resulted in the formation of SRC tumors in the lungs. Since this was observed even in the animals that had their legs amputated within minutes of transplantation, the observed SRC lung tumors were considered to result from colonization of SRC tumor cells into the lungs, as opposed to representing true lung metastases. The animals were euthanized 35 days post tumor induction, and the tumor tissues were frozen in liquid nitrogen immediately after excision and stored at -80°C or placed in paraformaldehyde for histology. For histology, tumors were graded based on previously described characteristics of human chondrosarcoma [13,14]

Normal rat cartilage was obtained from femoral head cartilage of 37-40 day old male Sprague-Dawley rats as previously described [12].

### Total RNA isolation

Total RNA was obtained from frozen tissues using TRIZOL reagent (Life Technologies, Inc.). Total RNA was treated with DNase (Promega #M6101), and subsequently treated with Proteinase K (Promega # 9PIV302). Total RNA was further purified using RNeasy kit (Qiagen), and then used for subsequent reactions.

### Sodium bisulfite treatment of DNA

Genomic DNA was obtained by digestion with proteinase K (Qiagen) followed by phenol/chloroform extraction, and was subjected to sodium bisulfite treatment to modify unmethylated cytosines to uracil using the 'CpGenome™DNA Modification Kit' (Chemicon International, CA).

### Pyrosequencing primer design

Satellite 1 primers were designed as previously described [9]. Briefly, the rat genome sequence (rn4/version 3.4, Nov. 2004), and the annotation for repetitive elements, were obtained from the UCSC Genome Database. Satellite 1 sequences were extracted and subjected to *in silico *bisulfite conversion based on their genomic coordinates in the UCSC database. Full-length Satellite 1 sequences were identified and used for alignment to generate a Satellite 1 nucleotide base matrix. A region within Satellite 1 sequence with dense CpG dinucleotides was selected for PCR primer design. An electronic PCR was performed with the primers designed for rat Satellite 1 sequences. A minimum of 137 distinct Satellite I elements were predicted to be targeted in PCR reactions with the primer set designed. Using a single sequencing primer, a total of 3 CpG dinucleotides were sequenced for each Satellite 1 element targeted. The global methylation data generated was derived from a minimum of 411 CpG dinucleotides in Satellite elements.

### SAGE library construction and data analysis

Poly (A)+ RNA was isolated from total RNA using mRNA DIRECT Kit (Dynal) according to manufacturer's instructions. The poly(A)+ RNA and a biotinylated oligo d(T) primer were used for cDNA synthesis according to a previously described method[15]. SAGE was carried out as previously described [10].

Approximately 100,000 tags were derived from each SAGE library. The initial sequencing files from each SAGE library were processed with SAGE2000 (Johns Hopkins University). For all analyses, each SAGE library was normalized to 100,000 tags. SAGE libraries were annotated using SAGEmap (http://www.ncbi.nlm.nih.gov/projects/SAGE/). Mitochondrial tags were identified using previously described annotation[16]. Genespring was used to perform hierarchical clustering, and to graphically represent the SAGE data. See Additional file 1 for complete SAGE data, Additional file 2 for the list of differentially expressed SAGE tags, and Additional file 3 for the SAGE tag list of unique expression profiles.

Gene expression comparisons between SRC tumors: Only genes with significantly different gene expression were included in each analysis (z > 1.96; see "statistical analysis"). SAGE tags also needed to have an expression level of at least 25 in one tissue to be included in the analysis. For the gene expression comparisons between SRC tumors, condition trees illustrate the relationship between the SAGE libraries with respect to the set of differentially expressed genes.

All SAGE data is GEO compliant. The raw SAGE data has been submitted to the GEO database. GSE1517 is the accession number of the Swarm rat chondrosarcoma SAGE data.

### Real-Time quantitative PCR

Total RNA was isolated using Trizol; RNA was treated with TURBO RNase-free DNase (Ambion Cat# AM1907). Total RNA (1 μg) was used to make cDNA with the iScript cDNA Synthesis kit (BioRad). Real time PCR was performed with the iQ SYBR Green Supermix (BioRad), and rat specific primers. Real time PCR primers were designed with Beacon Designer 6.0 (Premier Biosoft International; Palo Alto CA). Thymosin-β4 primers were as follows: Forward: CACATCAAAGAATCAGAACTAC; Reverse: TCTCAATTCCACCATCTCC. C-fos primers were as follows: Forward: ACCACGACCATGATGTTC; Reverse: AAGTTGGCACTAGAGACG. For SYBR green PCRs, 18S-rRNA was used as a reference gene [17]. The 18 S rRNA primers were as follows: Forward: GGGAGGTAGTGACGAAAAATAACAAT; Reverse: TTGCCCTCCAATGGATCCT.

To measure the expression of the transgene construct, primers were designed for the IRES (Internal Ribosome Entry Sequence). The IRES sequence is present in all expression vectors used within this report. The primer sequence; MSCV-IRES-F: TCTGTAGCGACCCTTTGC and MSCV-IRES-R: TTCCACAACTATCCAACTCAC. 18S-RNA was used as a reference gene for the analysis of transgene expression.

The Pfaffl method was used to calculate the normalized gene expression [18]. For each real time PCR analysis the individual sample being examined was used as the test sample in the Pfaffl method. The calibrator sample, for the Pfaffl method, was an equal mixture of cDNA from rat normal cartilage, SRC tumor, and/or SRC cell line. All real time qPCR results are displayed as a ratio of the target gene relative to the reference gene, in a specific test sample, compared to the expression of the target gene relative to the reference gene in the calibrator sample.

### Thymosin-β4 and c-fos overexpression

Two vectors were made for the overexpression experiments: MSCV-Thyβ4-I-Puro and MSCV-cfos-I-Puro. The expressions of Thyβ4 and c-fos were driven by the retroviral LTR, and the expression of the Puromycin resistance gene was controlled by the IRES sequence.

The rat thymosin-β4 coding sequence was PCR amplified from a rat normal cartilage cDNA library clone (UI-R-DY1-cns-1-12-0-UI) using the following primers: Forward: CTCTGAGCAGGAATTCTCTCCTTGTTCGCCCAGCTC, and Reverse: CTCAGTCAGTCTCGAGTGCCCTGCCTTCTCTGACTG. The resulting thymosin-β4 PCR product was digested with EcoRI and XhoI. The digested PCR product was ligated to an EcoRI-XhoI digested MSCV-I-Puro vector.

The rat c-fos coding sequence was PCR amplified from a Swarm rat chondrosarcoma cDNA library clone (UI-R-DZ0-crj-j-07-0-UI) using the following primers: Forward: TCTACCCCTGGAATTCTCGCCGAGCTTTGCCCAAAC, and Reverse: CTCAGTCAGTCTCGAGTGCCCTGCCTTCTCTGACTG. The resulting c-fos PCR product was digested with EcoRI and XhoI. The digested PCR product was ligated to an EcoRI-XhoI digested MSCV-I-Puro vector.

A murine stem cell virus was prepared by transfecting 293T cells with three plasmids; pMSCV-I-Hygro vector (for control cells: pMSCV-I-Hygro; for thymosin-β4: MSCV-Thyβ4-I-Puro; for c-fos expression MSCV-cFos-I-Puro), pEQ-Pam3(-E) (which encodes retroviral gag and pol) and pSRα-G (which encodes glycoprotein G from Vesicular Stomatitis Virus) [19]. Forty-eight hours post-transfection, media containing retroviral vector was collected, aliquoted, frozen, and stored at -80°C. This vector was then used to transduce the Swarm rat chondrosarcoma cell line (SRC-LTC (Long Term Culture) [20], [obtained from Jeff W. Stevens, University of Iowa]), in the presence of 5 μg/ml polybrene on three successive days allowing the cells to recover in the media used, generally overnight. Transduced cells were selected by incubation with puromycin at a concentration of 3 μg/ml for 14 days. The overexpression of thymosin-β4 and c-fos was confirmed following puromycin selection.

### Cell culture conditions

SRC-LTC cells were cultured in DMEM high glucose (4.5 g glucose/ml) supplemented with 10% FBS and Penicillin/Streptomycin. Cells were plated at 2.5. × 10^4 ^cells with 6 ml of media in a 25 cm^2 ^T flask. Cells were grown until they became 80-90% confluent (6 days), and at this time the cells were trypsinized and split.

### Invasion assay

A Membrane Invasion Culture System (MICS) was used to measure the *in vitro *invasiveness of all SRC cell lines as previously described [21]. Briefly, a polycarbonate membrane with 10- μm pores was uniformly coated with a defined matrix. Both upper and lower wells of the chamber were filled with RPMI. For CTGF treatment, the RPMI was supplement with 50 ng, 100 ng, or 250 ng/mL of CTGF. Recombinant CTGF was obtained from PeproTech Inc (Rocky Hill, NJ) (C-terminal peptide; product# 120-19). SRC cells were seeded into upper wells at a concentration of 5 × 10^5 ^cells per well. After a 24-hour incubation in a humidified incubator at 37°C with 5% CO_2_, cells that had invaded through the basement membrane were collected, stained, and counted by light microscopy [22].

### Tumor inductions in nude mice and tissue processing

The SRC cells were grown until they were 80% confluent, the cells were then washed with PBS, and then cells were removed from the plate using TrypLE Express (GIBCO cat#: 12605-010) according to manufacturer's instructions. Following removal of SRC cells from plates, the cells were washed with PBS, centrifuged, and resuspended in PBS. 5 × 10^6 ^cells were injected subcutaneously into the lower lumbar region of four-week old nude mice (Males; Charles River, Strain code: 088). For the control group, the SRC -LTC-MSCV-I-Hygro (cells expressing the empty viral vector) were injected into 10 separate mice. For one experimental group, SRC-LTC- MSCV-Thyβ4-I-Puro cells were injected into 10 individual mice. For the other experimental group, SRC-LTC- MSCV-cFos-I-Puro cells were injected into an additional 10 individual mice.

Following the injection, the animals were monitored twice weekly for 30 days. After 30 days the animals were euthanized by CO_2 _gas inhalation followed by cervical dislocation. Immediately following euthanization, tumors and other tissues were frozen in liquid nitrogen or placed in paraformaldehyde for histologic examination.

Nude mice were selected for this study because previous experiments in our laboratory had demonstrated that the SRC-LTC cell line grew in nude mice without host rejection. Since the SRC-LTC was modified with a retrovirus, we wanted to reduce the chance that the tumor cells would be rejected by the host immune response. Previous experiments in our laboratory had also indicated that the subcutaneous injection of SRC-LTC cells (modified with retrovirus) into nude mice would lead to the formation of palpable tumors in 4 weeks. Subcutaneous injection of SRC-LTC cell line into rats resulted in slower tumor growth than those generated in nude mice. Palpable tumors were detected at 4 months in rats compared to less than 1 month in nude mice. To limit experimental time, and to prevent the rejection of tumor cells by the host, nude mice were selected for the transplantation experiments with SRC-LTC cells.

### Statistical analysis

Significant differences in tag count among SAGE tag libraries were determined using a Z-test (∝ = .05) [23]. When the significance level is set at 0.05, a z-value greater than 1.96 is considered as a statistically significant difference.

DNA methylation level data were analyzed with the analysis of variance (ANOVA) method and mean DNA methylation levels from three groups were compared with the normal rat cartilage group and the resulting p-values were adjusted using the Dunnett method. We used statistical software SAS 9.1 and R to conduct analysis and generate figures. We attributed statistical significance to p values ≤ 0.05 in all comparisons.

Significant pathways and their associated p-values were determined using the pathway-mapping program Ingenuity. The p-value associated with a specific pathway is a measure of its significance with respect to the genes in a given data set and a reference set of genes (containing all of the members of predetermined pathway). P-values were calculated using the right-tailed Fisher's Exact Test, and the purpose of this test is to analyze a data set to identify the pathways that contain more genes from a given data set than would be expected by random chance. Statistically significant, non-random association is attributed to p values < 0.05 (see Ingenuity for more detailed explanations of the statistical analysis; http://www.ingenuity.com/).

## Results

### Tumor transplantation site influences tumor phenotype

**Figure 1:**
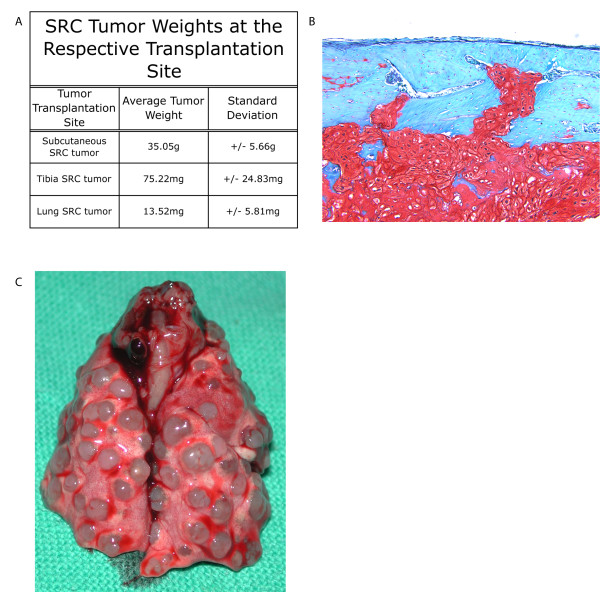
**Phenotype of the Swarm Rat chondrosarcoma varies based on tumor transplantation site**. (A)Tumor weight following transplantation into different sites. The average tumor weight and standard deviation are shown. The weight of the subcutaneous SRC tumor was calculated from 6 animals. The tibia SRC tumor weight was calculated based on tibia tumors isolated from 14 separate animals. The lung tumor weight represents the average weight of individual tumor nodules collected from 7 animals. The lung tumors were isolated from animals that had SRC tumor transplanted into the tibia (7 of 14 animals had lung tumors). (B)Transplantation of the SRC tumor into the tibia of Sprague-Dawley rats. Histologic micrograph at day 34 revealed increased tumor volume and invasion of the tumor into the bony cortex. Cells stained with safranin O and fast green. (C)SRC tumors detected in the lung of a rat that had the SRC tumor transplanted into the tibia. Note the presence of multiple SRC tumors. Lung tumors were detected in 50% of the animals that had the SRC tumor transplanted into the tibia. The number of lung SRC tumors in a single animal ranged from 1 to 54 (average = 10). The average size of the tumors was 2 mm. No lung tumors were detected in the animals with subcutaneous tumor transplants.

Tumors were initiated by transplanting SRC tumor cells subcutaneously or into the tibia of Sprague-Dawley rats. The tumors exhibited different growth and histologic characteristics depending on the tumor transplantation site. Approximately 3 weeks following subcutaneous transplantation, tumors were isolated and determined to have an average weight of 35.05 g (Figure 1A). Three weeks following tumor transplantation into the tibia the tumors weighed an average of 75.22 mg (Figure 1A). Transplantation of the SRC tumor into the tibia resulted in bone destruction and tumor invasion into the surrounding cortex of the bone (Figure 1B). In comparison, the subcutaneous SRC tumors did not invade into surrounding tissues. Therefore, as reported previously [8], the SRC tibia tumor was classified as a higher grade tumor than the subcutaneous (extraosseous) SRC tumors.

Tumor transplantation into the tibia also led to the formation of SRC tumors in the lungs of rats (50% of animals; Figure 1C). No SRC lung tumors were detected in the lungs of rats that had the SRC tumor transplanted subcutaneously.

Although 50% of the animals with the tibia SRC tumor also developed lung SRC tumors, the latter most likely resulted from colonization of tumor cells in the lungs rather than metastasis. As pointed out before, animals that had their leg amputated immediately following tumor transplantation did exhibit tumors in the lungs. Hence, we do not refer to the SRC lung tumor as a metastasis. Since chondrosarcoma does metastasize to the lungs in humans, we reason the inclusion of the SRC lung tumors in our analyses is justified in that it might provide relevant information relating to chondrosarcoma development and progression.

### Epigenetic analysis of SRC tumors

Epigenetic analyses were carried out to determine if there was a difference in the DNA methylation levels of the tumors that were initiated at different transplantation sites. The methylation level of cytosines in CpG dinucleotides of repetitive elements has been used as a marker for assessment of genome-wide levels of methylation [9,24]. Hence the Satellite 1 repetitive element was selected as a methylation marker in our study. Rat specific pyrosequencing assays were designed to examine the methylation of Satellite 1 sequences throughout the genome. Pyrosequencing was performed on DNA isolated from control tissue, rat normal (articular) cartilage (RNC), and on SRC tumor tissues derived from the different transplantation sites.

**Figure 2:**
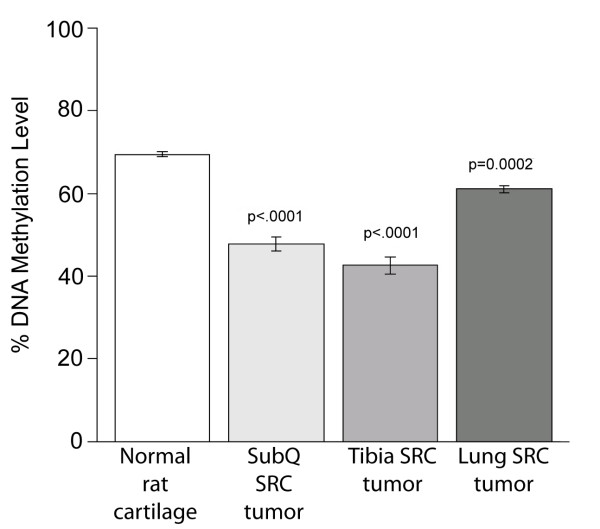
**Transplantation microenvironment influences DNA methylation in SRC tumors**. Pyrosequencing revealed that Satellite 1 DNA was hypomethylated compared to DNA from normal rat cartilage (control tissue). The satellite 1 DNA elements in the subcutaneous SRC tumor and the tibia SRC tumor were hypomethylated compared to the DNA in the lung SRC tumor. The graph illustrates the average Satellite 1 DNA methylation calculated from a pool of tissues from each transplantation site. For each transplantation site, tumor tissue was pooled from at least 10 separate animals. Bars represent the average DNA methylation % of technical replicates of the pooled tissue samples, and error bars represent the standard deviation of these replicates. P-values are indicated for comparisons between the specific sample and the "Normal rat cartilage" sample using Dunnett method in the analysis of variance (ANOVA). We attributed statistical significance to p values ≤ 0.05.

Pyrosequencing of rat satellite 1 revealed methylation differences between the SRC tumors and rat normal cartilage, as well as among the SRC tumors at different transplantation sites. Specifically, the SRC tumors exhibited a lower level of methylation than the rat normal cartilage (Figure 2). Amongst the SRC tumors, the subcutaneous tumor and the tibia tumor had lower Satellite 1 methylation levels than the lung tumor (Figure 2).

These results demonstrate that the Satellite 1 DNA is hypomethylated in SRC tumors compared to control tissue. Our results also indicate that the transplantation site can influence DNA methylation levels in SRC tumors. Since the observed differences in methylation level involved satellite 1 DNA sequences mapped throughout the genome, it is likely that the observed alterations in methylation may be indicative of other changes in methylation that might accompany tumor growth at different transplantation sites.

### SAGE library description

**Figure 3:**
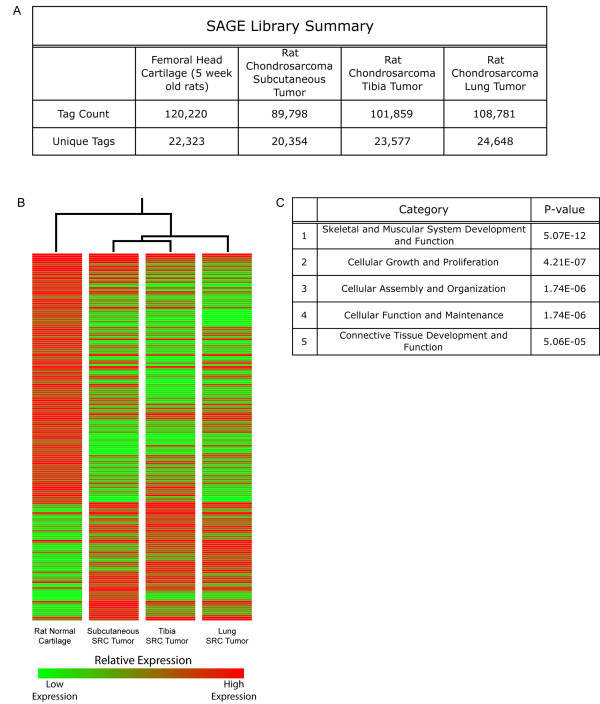
**Summary and analyses of SAGE libraries.** (A) Summary of SAGE tags generated from normal cartilage and the SRC tumors. The total number of SAGE tags and the number of unique tags are listed for each SAGE library. (B) Heat map displaying the differentially expressed genes between RNC and SRC tumor tissues. Rat normal (articular) cartilage has a unique expression profile when compared to the expression profiles of the SRC tumors. The changes in gene expression may represent critical differences between normal cartilage cells and chondrosarcoma, and they may also represent changes important for the development and progression of chondrosarcoma. Heat map displays the differentially expressed genes that were expressed at a level of at least 25 tags in one library. For complete gene list see Additional file 2. Color bar illustrates relative gene expression levels. (C) The list of differentially expressed genes was analyzed with the pathway-mapping program Ingenuity. The top five functional pathways and their corresponding p-values are displayed in table (see “Methods” for description of p-value).

Based on the aforementioned differences among the SRC tumors, we hypothesized that tumors would also exhibit significant differences in gene expression. To test this hypothesis, SAGE was used to generate gene expression profiles of the SRC tumors. SAGE profiles were generated for rat normal cartilage, the subcutaneous SRC tumor, the tibia SRC tumor, and the SRC lung tumor. Over 400,000 SAGE tags were generated for this analysis. The total number of SAGE tags sequenced and the number of unique tags in each library are shown in Figure 3A.

### Gene expression differences between normal cartilage and the SRC tumors

**Figure 4:**
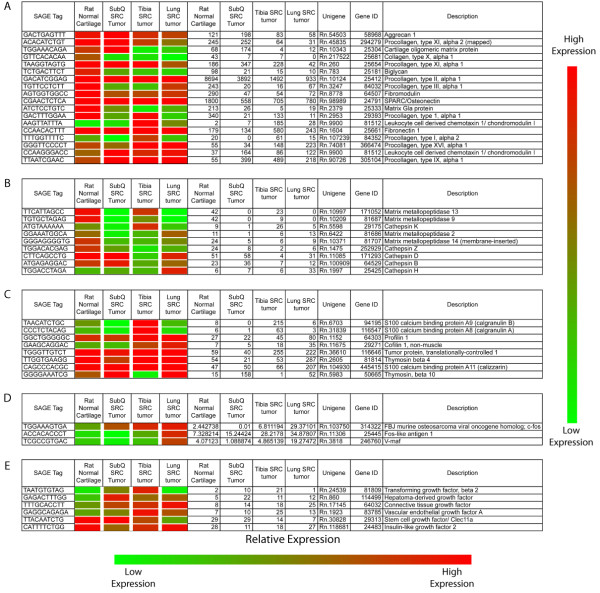
**SAGE reveals gene expression differences between the SRC tumors and normal cartilage as well as gene expression differences between SRC tumors**. (A) Expression of extracellular matrix genes. (B) Expression of extracellular matrix modifying proteases. (C) Expression of genes related to cell motility. (D) Expression of components of the AP-1 transcription factor complex. (E) Expression of growth factors. The Heat map represents relative gene expression. Actual expression values are listed to the right of the heat map.

The SRC tumors exhibited significantly different gene expression profiles compared to that of normal rat cartilage (control tissue), and these gene expression changes distinguish tumors from RNC (Figure 3B). Analysis of the differentially expressed genes revealed changes in several pathways that may be important to chondrosarcoma tumorigenesis. (Figure 3C). The most significantly altered pathway, "Skeletal and muscular system development and function", highlighted differences in gene expression that could directly impact the extracellular matrix of both tumor cells and surrounding host cells. Specifically, gene expression alterations were detected for structural extracellular matrix genes (Figure 4A) and for extracellular matrix modifying proteases (Figure 4B).

Changes to the extracellular matrix have prognostic value in chondrosarcoma. Decreased expression of specific extracellular matrix molecules, as observed with the SRC tumors compared to normal tissue (Figure 4A), has been associated with high grade human chondrosarcomas [25]. Increased expression of specific proteases has also been reported in human chondrosarcoma [26,27].

These results indicate that gene expression alterations in the SRC tumors include changes also observed in human chondrosarcoma, thus providing additional support to previous work demonstrating that the SRC tumor model resembles human chondrosarcoma [7].

### Transplantation site influences gene expression

Although most structural extracellular matrix genes were expressed at lower levels in the SRC tumors than in normal cartilage, closer analysis revealed changes in gene expression that were unique to the SRC tumor at each transplantation site (Figure 4A). Additionally, the expression of specific proteases varied among tumors. The observed alterations in the expression of proteases among SRC tumors may thus represent changes that are unique to each specific transplantation site (Figure 4B).

**Figure 5:**
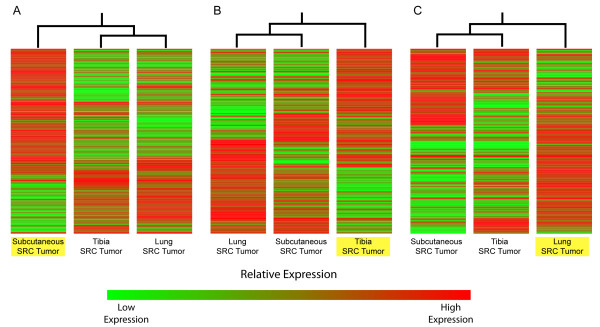
**Tumor transplantation site significantly alters the gene expression profiles of the SRC tumors**. Since the SRC tumors at different transplant sites originated from the same source tumor, the unique SRC gene expression profiles at each transplant site are likely a result of interactions in the microenvironment between tumor cells and host cells. For each gene expression comparison (A-C), a different tumor was selected as a "reference sample"(highlighted in yellow). The gene expression of the "reference sample" was then compared with the gene expression profiles of the other tumors. For each comparison, the upregulation/downregulation refers to the status of the genes in the "reference sample" compared to the tumor samples. (A) Differential gene expression between the Subcutaneous SRC tumor (reference sample; highlighted in yellow) and SRC tumors at the other transplantation sites (200 genes upregulated; 107 genes downregulated in the Subcutaneous SRC tumor). (B) Differential gene expression between the Tibia SRC tumor (reference sample; highlighted in yellow) and the SRC tumors at the other transplantation sites (106 genes upregulated; 108 genes downregulated in the tibia SRC tumor). (C) Differential gene expression between the Lung SRC tumor (reference sample; highlighted in yellow) and the SRC tumors at the other transplantation sites (157 genes upregulated; 73 genes downregulated in the lung SRC tumor). Color bar illustrates relative gene expression levels. See "Methods" for additional description (Gene expression comparisons between SRC tumors), and see Additional file 3 for complete data set and annotation of the genes presented within this figure.

Further analysis of the SAGE data revealed that the gene expression profiles of the tumors are unique to their transplantation sites (Figure 5). Although each SRC tumor originated from the same tumor source, significant gene expression differences were detected among the SRC tumors. Characterization of these differences revealed changes in the expression of genes involved in regulating "Cellular Assembly and Organization" (Figure 3C). Several genes related to cell motility were upregulated in both the tibia and lung SRC tumors (Figure 4C). The altered expression of cell motility-related genes suggests that both the tibia and the lung microenvironments may promote changes in the actin cytoskeleton, which in turn may have a direct impact on the invasiveness of SRC cells.

### Endogenous thymosin-β4 expression in the SRC tumors

**Figure 6:**
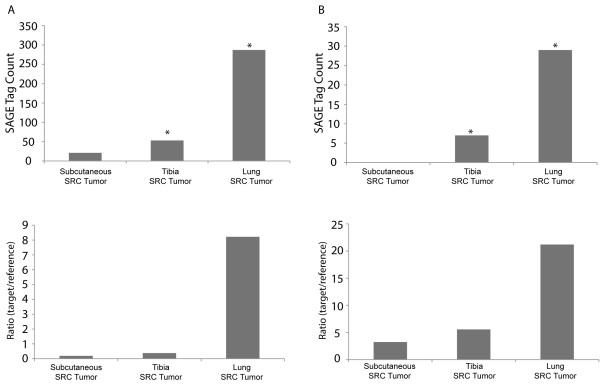
**SAGE and quantitative RT-PCR confirm the expression of thymosin-β4 and c-fos in the SRC tumors**. (A) Thymosin-β4 expression in the SRC tumors (SAGE analysis top panel; quantitative RT-PCR bottom panel). (B) C-fos expression in the SRC tumors (SAGE analysis top panel; quantitative RT-PCR bottom panel). SAGE and RT-PCR identified similar gene expression patterns for both thymosin-β4 and c-fos. Note the increased expression of thymosin-β4 and c-fos in the tibia and lung SRC tumors. The graphical bars in the RT-PCR figure represent the average expression ratio calculated on RNA that was collected from pooled tumor tissue. For RT-PCR at each transplantation site, tissue was pooled from at least 10 separate tumors. SAGE data was normalized to 100,000 tags/library for analysis. In the SAGE graph, "*" indicates that the expression levels of a specific sample are significantly different relative to the "Subcutaneous SRC tumor" sample (z > 1.96).

It is noteworthy that one of the genes identified in the cell motility pathway, thymosin-β4, is significantly upregulated in the tibia and lung SRC tumors (Figure 6A). Thymosin-β4 is thought to play a role in the cytoskeletal organization of chondrocytes [28], and overexpression of thymosin-β4 may influence tumorigenicity and metastasis [29].

### Endogenous c-fos expression in the SRC tumors

The second most significantly altered pathway was "Cellular Growth and Proliferation"(Figure 3C). More detailed examination of this pathway identified several differentially expressed genes that are components of the AP-1 transcription factor complex (Figure 4D). AP-1 is a potent transcription factor that has multiple functions in tumor cells [30]. One particular component of AP-1, c-fos, was differentially expressed in both the tibia and lung SRC tumors (Figure 6B). Expression of c-fos has been investigated in human chondrosarcoma [31]. Overexpression of c-fos leads to the development of chondrogenic tumors [32], and c-fos activity has been associated with increased invasiveness of chondrosarcoma cells [33].

### Growth factor expression in the SRC tumors

Gene expression alterations in the SRC could promote additional expression changes in the SRC cells and/or may lead to altered expression in the surrounding host cells. For example, changes in growth factor expression were detected in the SRC tumors (Figure 4E). These growth factors could be secreted into extracellular matrix where they have the potential to interact with tumor and/or host cells. Taken together, these results indicate that the tumor transplantation site has a significant impact on the gene expression profile of the SRC cells. These analyses provide insight into the interaction between the SRC cells and the transplantation site, as well as to the specific pathways that may contribute to SRC tumorigenesis.

### Functional analysis of differentially expressed genes

#### Overexpression of thymosin-β4 and c-fos

**Figure 7:**
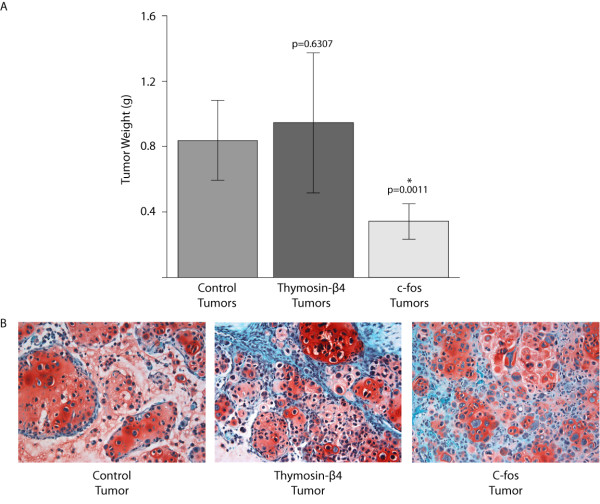
**Tumor weight following induction of subcutaneous tumors with SRC cells overexpressing either thymosin-β4 or c-fos**. Control tumors were initiated with SRC cells that express the MSCV viral vector with no insert (pMSCV-I-Hygro vector). C-fos tumors were initiated with SRC cells that overexpress c-fos (MSCV-cfos-I-Puro). Thymosin-β4 tumors were initiated with SRC cells that overexpress thymosin-β4 (MSCV-Thyβ4-I-Puro). Overexpression of c-fos resulted in the formation of tumors that were significantly smaller than control tumors. The bar represents the average invasion indices of biologic replicates, and the error bars represent the standard deviation of the biologic replicates. '*' Indicates that the tumor weight is significantly different than the tumor weight of the control tumors (p-value <0.05 considered significant). n = 10 for control tumors and c-fos tumors. n = 9 for thymosin-β4 tumors (one animal died prematurely and was found to have multiple chondrosarcoma lung metastases). See Additional file 4 for complete list of tumor weights. (B) Photomicroscopy of histological sections obtained from SRC tumors (20 × magnification). Tumors induced from control cells (SRC cells expressing the empty viral vector), and from SRC cells overexpressing either Thymosin-β4 (Thymosin-β4 tumor) or c-fos (C-fos tumor). Approximately 30 days following tumor induction animals were sacrificed and tumors were removed for histology. Sections representative of each tumor are shown. All tumors were classified as histologic grade II chondrosarcomas. The SRC cells are stained with Safranin O (red).

Based both on their differential expression (Figure 6) and on their potential role in tumorigenesis, thymosin-β4 and c-fos were selected for additional analyses. Thymosin-β4 and c-fos were independently overexpressed in a SRC cell line and the cell lines were used to induce subcutaneous SRC tumors (Figure 7). Control tumors were generated with SRC cells expressing an empty viral vector, while the tumors in the experimental groups were induced by injection of SRC cells overexpressing either c-fos or Thymosin-β4. Histologically, the tumors were classified as grade II chondrosarcomas (Figure 7B), but certain phenotypic differences were observed between the tumors.

Overexpression of thymosin-β4 resulted in the formation of the largest SRC tumors (Additional file 4). However, the size of the thymosin-β4-overexpressing-tumors varied among animals and the average tumor weight was not statistically significantly different from that of tumors derived from control cells (Figure 7A). Although thymosin-β4 tumors did not exhibit a statistically significant difference in size relative to the control tumors, it should be noted that one of the mice (with the thymosin-β4 tumor) died before the end of the *in vivo *experiment (n = 10; 1 mouse died; see Additional file 4). Histologic analysis of this animal revealed multiple lung chondrosarcoma micrometastases. The finding of lung metastasis in this animal is particularly intriguing due to the fact that lung metastases have previously not been detected following subcutaneous injection of SRC cells.

C-fos overexpression resulted in the formation of tumors that were significantly smaller than control tumors (Figure 7A and Additional file 4). Micrometastasis was not detected in any animals with c-fos overexpressing tumors.

### CTGF and the SRC cells

**Figure 8:**
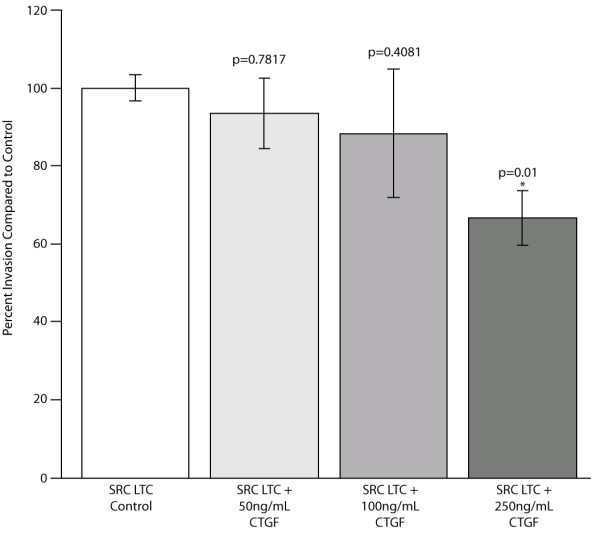
**CTGF treatment decreases the invasiveness of SRC cells**. Invasiveness was measured in control SRC cells (SRC Control) and in SRC cells treated with CTGF (50, 100, and 250 ng/mL) at the start of the invasion assay. Twenty-four hours later, the invasiveness was calculated for all samples and the results are displayed as experimental sample compared to the untreated control SRC cells (100% invasion). The bar represents the average invasion indices of biologic replicates, and the error bars represent the standard deviation of 3 biologic replicates.'*' Indicates values that are significantly different than the "SRC Control" sample (p < .05).

Growth factor expression varied with the tumor transplantation site (Figure 4E), but the functional consequences of these changes are unknown. One growth factor, CTGF (Connective Tissue Growth Factor), was selected for further analysis because of its differential expression and previously reported altered expression in several cancers (including chondrosarcoma) [34-37]. To test the influence of CTGF, the invasiveness of the SRC cells was examined following incubation with varying concentrations of CTGF. The lower doses of CTGF (50 and 100 ng/mL) did not significantly alter the invasiveness compared to that of control cells, but a higher concentration of CTGF resulted in a significant decrease (30%) in SRC invasiveness (Figure 8).

## Discussion

Tumor microenvironment is an important factor that can influence the malignancy of SRC tumors [8]. Previous studies have characterized the histological changes that accompany SRC tumor growth at different transplantation sites [8]. However, little is known about the gene expression changes that underlie the histological changes. To address this issue, we examined the epigenetic and gene expression changes following SRC growth at different tumor transplantation sites. Epigenetic and gene expression changes were detected between the SRC and normal tissue, and additional analysis revealed gene expression changes among SRC tumors grown at different transplantation sites. Closer examination of differentially expressed genes and subsequent functional analysis provided insight into the involvement that specific genes may have in chondrosarcoma tumorigenesis.

Subcutaneous SRC tumors ultimately produced the largest tumors (Figure 1A). However, as it is the case with human chondrosarcoma, tumor size does not necessarily correlate with histologic grade [38]. The tibia tumor displayed increased tumor infiltration and bone destruction over time (Figure 1B). Such invasive behavior was not observed in the subcutaneous SRC tumors. These findings suggest that the tibia SRC tumors have increased invasive characteristics relative to the subcutaneous SRC tumors. These findings are also in agreement with previous findings demonstrating that SRC transplantation in the tibia results in tumor infiltration [7], and changes in the malignancy of the SRC cells [8].

Epigenetic analysis of the SRC tumors revealed that the SRC tumors are hypomethylated compared to normal rat cartilage. The epigenetic analysis also demonstrated that the tumor transplantation site can influence the DNA methylation levels of the SRC tumors. This result supports previous observations suggesting that the microenvironment may modulate epigenetic events in solid tumors [39]. Although the impact of hypomethylation on the SRC cells is largely unknown, DNA hypomethylation has been documented to occur in several types of cancer [40]. Most importantly, previous studies have demonstrated that DNA hypomethylation may have a significant impact on tumorigenesis [9,41,42].

In addition to affecting DNA methylation, the transplantation sites had a considerable impact on the global gene expression profiles of SRC tumors. Such expression changes provide some insight to the molecular mechanisms underlying growth and progression of SRC tumors.

Furthermore, the SRC tumors exhibit a gene expression profile that is significantly different from that of normal rat cartilage, including a major decrease in the expression of several extracellular matrix molecules (Figure 4A). Studies of human chondrosarcoma have indicated that expression of collagen type II and aggregan are indicative of a mature neoplasm with low recurrence and low chance for metastasis [5]. A decrease in the expression of extracellular matrix molecules, as observed in SRC tumors (Figure 4A), may represent a change in the nature of the SRC tumors from a mature neoplasm to a more aggressive less differentiated neoplasm.

The SRC tumors also expressed several matrix metalloproteases and cathepsins (Figure 4B). The expression of these proteases varied depending on the SRC transplantation site, but since these proteases alter the extracellular matrix, their expression may have a significant impact on tumor progression. Altered expression of matrix metalloproteases and cathepsins has previously been reported in human chondrosarcoma [26,27], and their expression may play an important role altering the extracellular matrix and promoting tumor invasion. Cathepsin K, for example, is thought to play a role in human chondrosarcoma progression [26], and it is expressed at highest levels in tibia SRC tumors (Figure 4B). Cathepsin K is a protease that has the ability to degrade collagen type I [43], a major component of bone. The expression of cathepsin K could explain the degradation of bone that is observed with the tibia SRC tumors (Figure 1B). The expression of cathepsin K combined with the expression of other proteases may lead to extracellular matrix degradation and subsequent SRC tumor progression.

Further analysis of the SAGE data revealed additional gene expression changes that may also contribute to chondrosarcoma tumorigenesis. Both the tibia SRC tumor and the lung SRC tumor displayed elevated levels of mRNAs regulating the actin cytoskeleton (Figure 4C), and the SRC tumors also displayed elevated levels of transcripts controlling cellular growth and proliferation (Figure 4D). Overexpression of a cell motility related gene, thymosin-β4, did not produce tumors that were significantly larger than control tumors (Figure 7). However, overexpression of thymosin-β4 resulted in chondrosarcoma lung metastasis and subsequent death in one animal. Since tumor size does not correlate with tumor malignancy in human chondrosarcoma [38], the fact that thymosin-β4 overexpressing tumors were not larger than control tumors does not preclude the hypothesis that they may have a greater malignant potential. It should be emphasized, however, that although intriguing this observation has to be considered with great caution since it is based on a single animal.

Although the function of thymosin-β4 in the SRC is not known, high levels of thymosin-β4 have been detected in human chondrosarcoma (National Cancer Institute: SAGE Genie database). Overexpression of thymosin -β4 has previously been shown to regulate motility and invasiveness in fibrosarcoma [29], and reports in melanoma suggest that thymosin-β4 can stimulate metastasis through the activation of cell migration and angiogenesis [44]. The ability of thymosin-β4 to increase tumor cell motility is thought to be related to its function in the regulation of the actin cytoskeleton [45], but thymosin-β4 may have other functions in the SRC cells. Thymosin-β4 can be secreted into the extracellular matrix [46], and extracellular stimulation with thymosin-β4 may lead to increased Ap-1 activity.

C-fos is a component of the AP-1 transcription factor complex, and AP-1 is thought to play multiple roles in tumorigenesis [30]. Overepxression of c-fos resulted in the formation of tumors that were significantly smaller than control tumors (Figure 7), which was not expected. However, this result suggests that either directly or indirectly c-fos' activity does seem to influence tumor growth. C-fos and AP-1 signaling have been associated with chondrosarcoma development [47], and c-fos signaling may influence the invasiveness of human chondrosarcoma cells [48,49].

The signaling mediators and effectors of c-fos/AP-1 interactions may vary depending on the microenvironment of the transplantation site, and they may include growth factors, cytokines, and/or other signaling molecules. The expression analysis conducted in this study revealed significant changes in growth factor expression amongst the SRC tumors (Figure 4E). Most noteworthy, VEGF, TGFB2 and CTGF, all of which are expressed in human chondrosarcoma, were also found to be expressed in SRC tumors. The potential function of these growth factors range from cell motility, to cell growth, to angiogenesis [50-52]. In this study, incubation with CTGF led to a decrease in the invasiveness of the SRC cells (Figure 8). Although this result may appear counterintuitive to a role for CTGF in tumor progression, CTGF has recently been shown to enhance cell adhesion of a human chondrosarcoma cell line through interaction with fibronectin (also expressed in the SRC cells; Figure 4A) [53]. Regardless, the fact that incubation with CTGF affected invasion, albeit in the opposite direction to that expected, clearly points to its activity being directly or indirectly associated with invasiveness in the SRC tumor model. Accordingly, we speculate that through interaction with fibronectin, CTGF may promote adhesion and thus negatively affect motility. CTGF is expressed in normal lung cells [54,55], and an intriguing hypothesis is that CTGF may play a role in the adhesion of tumor cells in the lung. In addition to a role in cellular adhesion, CTGF has previously been shown to influence cell proliferation and angiogenesis [56,57], but additional experiments are needed to determine if CTGF affects these pathways in the SRC.

The mode of growth factor induction in the SRC tumors is not known, but growth factor induction could be mediated through AP-1 signaling. Binding sites for AP-1 have been identified in the promoter region of CTGF [58] and TGFβ-2 [59]. AP-1 is capable of activating the IGF-2 promoter [60], and the expression of VEGF has previously been shown to be mediated through AP-1 [61]. The induction of growth factors in the SRC may, in part, be regulated by AP-1. Alternatively, changes in AP-1 expression may also be influenced by growth factor expression [62-64].

## Conclusions

Taken together, these experiments highlight the importance of the tumor microenvironment in SRC tumorigenesis. Transplantation of a SRC tumor into different microenvironments in the rat resulted in phenotypic changes in the tumor. The changes in the phenotype were accompanied by alterations in the transcriptome and in the epigenome. DNA methylation patterns changed following tumor transplantation, indicating that the transplantation site can affect the DNA methylation of the SRC tumors. Despite originating form the same tumor source, the SRC tumors also displayed expression profiles unique to their transplantation sites. Subsequent functional analysis shed some light into the mechanisms of SRC tumorigenesis, and suggested that thymosin-β4 may contribute to the malignancy of SRC tumors.

Further research is needed to examine the function of thymosin-β4 in chondrosarcoma, and to identify factors that control its expression. For example, the promoter region of the human thymosin-β4 gene contains a CpG island [65], which indicates that DNA methylation could play a role in the regulation of thymosin-β4. Additionally, experiments are needed to determine how biologic signaling at the transplantation site affects DNA methylation, and to determine if these changes in DNA methylation have an effect on SRC tumorigenesis.

## List of abbreviations

SRC: Swarm rat chondrosarcoma; SAGE: Serial analysis of gene expression; CTGF: Connective tissue growth factor.

## Competing interests

The authors declare that they have no competing interests.

## Authors' contributions

CAH designed cellular and molecular experiments, constructed the SAGE libraries, carried out *in vivo *and *in vitro *experiments, and drafted the manuscript. JWS and JAM contributed to the design of the *in vivo *and *in vitro *SRC experiments and carried out transplantation experiments. HX contributed to the design and analysis of the pyrosequencing experiments. EAS assisted with the design and implementation of the invasion assays. EFV, HA, STS, JB, DW, SM MFB, TLC, and MJCH participated in the design of the study and revised the manuscript. MBS played an integral role in design of the study, study coordination, and critical manuscript revision. All authors read and approved the final manuscript.

## Pre-publication history

The pre-publication history for this paper can be accessed here:

http://www.biomedcentral.com/1471-2407/10/471/prepub

## Supplementary Material

Additional file 1 **Gene expression data generated with the SAGE experiments**. The SAGE data is presented as a table. The first row of the table describes each column of the table. Each subsequent row corresponds to a single SAGE tag. Each tag is identified by its 10 base-pair nucleic acid sequence. The adjacent columns provide the expression value for each tag in a given SAGE library. The raw expression data and the normalized expression values are given for each SAGE library (for the normalized data the tags were normalized to 100,000 tags/library). For each SAGE tag, the Unigene number and gene name description are given if known. Click here for file

Additional file 2 **Differentially expressed SAGE tags**. Complete list of differentially expressed genes obtained from the comparison of "Rat Normal Cartilage" vs. all 3 SRC SAGE libraries ("Subcutaneous SRC tumor", "Tibia SRC tumor", and "Lung SRC tumor"). The criteria for section was as follows: z-value > 1.96 (for differential gene expression) and expression of at least 25 tags in one SAGE library. The data is presented as a table. The first row of the table describes each column of the table. Each subsequent row corresponds to a single SAGE tag. Each tag is identified by its 10 base-pair nucleic acid sequence. The adjacent columns provide the expression value for each tag in a given SAGE library. For each SAGE tag, the Unigene number and gene name description are given if known. Click here for file

Additional file 3 **SAGE tag list of unique expression profiles**. The complete list of differentially expressed genes for the following comparisons are presented in Appendix C: "Subcutaneous SRC tumor" vs. "Tibia SRC tumor" and "Lung SRC tumor", "Tibia SRC tumor" vs. "Subcutaneous SRC tumor" and "Lung SRC tumor", and "Lung SRC tumor " vs. "Subcutaneous SRC tumor" and "Tibia SRC tumor". The criteria for selection was as follows: z-value > 1.96 (for differential gene expression) and expression of at least 25 tags in one SAGE library. The first row of the table describes each column of the table. Each subsequent row corresponds to a single SAGE tag. Each tag is identified by its 10 base-pair nucleic acid sequence. The adjacent columns provide the expression value for each tag in a given SAGE library. For each SAGE tag, the Unigene number and gene name description are given if known. Click here for file

Additional file 4 **Summary of subcutaneous tumor weight following transplantation of SRC cells that overexpress thymosin-β4 or c-fos**. Tumors harvested 33 days following subcutaneous tumor transplantation. Tumor weights are reported for control tumors, Thymosin-β4 tumors, and c-fos tumors. Click here for file
